# Supplementary material for: The synergy of damage repair and retention promotes rejuvenation and prolongs healthy lifespans in cell lineages
Source: PLoS Comput Biol. 2020 Oct 12;16(10):e1008314. doi: 10.1371/journal.pcbi.1008314 (PMC7598927; doi:10.1371/journal.pcbi.1008314)
Supplement: S1 Text — Detailed information about the single cell model as well as the non-dimensionalisation of it. (PDF) [file pcbi.1008314.s009.pdf]

The synergy of damage repair and retention promotes rejuvenation and prolongs healthy lifespans in cell lineages

S1 Text : Model construction and non-dimensionalisation

Barbara Schnitzer [1], Johannes Borgqvist [1], Marija Cvijovic [1*]

[1] Department of Mathematical Sciences, University of Gothenburg, Sweden
[*] corresponding author: Marija Cvijovic, marija.cvijovic@chalmers.se

model	term	description and biological justification
$\dot{\mathbf{P}}(\mathbf{t})$	$\mu P(t)\left(g - \frac{D(t)}{D_c}\right)$	Cell growth is modelled by the synthesis of functional proteins. μ is the growth rate of an exponential growth mediated by the uptake of key nutrients like sugars and nutrient compounds [1]. μ is assumed to be constant corresponding to a continuous inflow of resources, as it would be for instance in a microfluidics device. We added an additional unitless factor $g - \frac{D(t)}{D_c} \in [g-1, g]$ to account for the effect of damage on the effective growth rate of the cell which declines with age [2–4]. Thus, $g > 1$ is denoted as growth factor and determines the decline of the growth rate. The closer the cell gets to the amount of damage that leads to cell death D_c the slower it can grow.
	$-k_1 P(t)$	Functional proteins get damaged with a constant rate k_1 [4–6]. A damaged protein can for instance be misfolded or oxydised [7]. We assume that by these modifications proteins get totally dysfunctional for the cell.
	$+k_2 \frac{R D_c}{\pi} \sin\left[\frac{\pi}{R}\frac{D(t)}{D_c}\right]$	Cells have an extensive protein quality control system to transform back damaged to functional proteins by refolding or degradation and new synthesis by so-called chaperones and the ubiquitin-proteasome system [8–10]. We assume that this happens with a repair rate k_2. Since however also the protein quality control system suffers from too much damage [11, 12], we chose a non-linear term dependent on how close the cell is to cell death $\frac{D(t)}{D_c}$. This effect is reflected in the repair capacity R that sets how much the effective repair declines with age (see S2 Text for more details). The repair term, and in particular the repair capacity R, cannot explain mechanistic details underlying the repair machinery of yeast cells but can help to compare and understand the consequences of specific repair strategies.

model	term	description and biological justification
$\dot{\mathbf{D}}(\mathbf{t})$	$+k_1 P(t)$	Functional proteins get damaged with a constant rate k_1 [4–6]. A damaged protein can for instance be misfolded or oxydised [7]. We assume that by these modifications proteins get totally dysfunctional for the cell.
	$-k_2 \frac{R D_c}{\pi} \sin\left[\frac{\pi}{R}\frac{D(t)}{D_c}\right]$	Cells have an extensive protein quality control system to transform back damaged to functional proteins by refolding or degradation and new synthesis by so-called chaperones and the ubiquitin-proteasome system [8–10]. We assume that this happens with a repair rate k_2. Since however also the protein quality control system suffers from too much damage [11, 12], we chose a non-linear term dependent on how close the cell is to cell death $\frac{D(t)}{D_c}$. This effect is reflected in the repair capacity R that sets how much the effective repair declines with age (see S2 Text for more details). The repair term, and in particular the repair capacity R, cannot explain mechanistic details underlying the repair machinery of yeast cells but can help to compare and understand the consequences of specific repair strategies.

Table 1: Description of the ODE model illustrated in Fig 1A.

In addition to the ODE model in Table 1 there are two discrete events, cell division and cell death, in the single-cell model described in Table 2. Cell division has been modelled in the same way as in previous replicative ageing models [4–6, 13].

model	term	description and biological justification
**division**	$P(t_{div}) = P_c$	A cell divides at time t_{div} when it reaches a critical amount of intact proteins P_c [4–6] motivated by a critical cell volume that was proposed to be correlated to the initiation of cell division in yeast cells [14].
	distribution of $D(t_{div})$	The size proportion s describes the fraction of proteins that stays in the mother cell, interpretable as the size or volume symmetry of the cell division. To begin with, we assumed that proteins are diffusing with similar diffusion constants over the whole cell volume that are moreover sufficiently small that the equilibrium between mother and bud is reached fast in the timescale of a cell division, in accordance with [15–18], leading to similar protein concentrations in mother and bud (not necessarily the absolute number of proteins if $s \geq 0.5$). In that case we can estimate the number of damaged proteins that stay in the mother ($sD(t_{div})$) and in the bud ($(1-s)D(t_{div})$) only by the size proportion s. We added retention by a retention factor $0 \leq re \leq 1$, that describes the effect of various active and passive retention mechanisms together [3, 15–25] and introduces an asymmetry in the damaged components between mother and daughter cell. As a consequence, the mother is loaded with an additional amount of damage ($+re(1-s)D(t_{div})$), in favour of the daughter cell that in contrast contains less damage ($-re(1-s)D(t_{div})$). For $re = 1$ all damaged proteins are retained in the mother while the daughter stays completely damage-free. For $re = 0$ the previously described scenario where only s is crucial takes place. Consequently, we can model the degree of damage asymmetry caused by active and passive retention with the retention factor re.

model	term	description and biological justification
	distribution of $P(t_{div})$	Similar to the distribution of $D(t_{div})$, the corresponding distribution of $P(t_{div})$ to the mother $(sP(t_{div}))$ and the daughter cell $((1-s)P(t_{div}))$ is determined by the size proportion s under the same assumptions as before. If retention loads the mother with more damage it at the same time loses functional proteins $(-re(1-s)D(t_{div}))$ in order to assure mass conservation. In that way the mother contains s times the total amount of proteins after cell division. Accordingly, the daughter is loaded with additional functional proteins $(+re(1-s)D(t_{div}))$ to assure that the daughter cell is associated with $(1-s)$ times the total amount of proteins.
**death**	$D(t_d) = D_c$	If a cell has too many damaged proteins it cannot maintain its functionality and enters cell-cycle arrest before ultimate cell death [26, 27]. In the replicative ageing model a cell therefore dies at time t_d when it reached a critical amount of damaged proteins D_c [4].

Table 2: Description of cell division and cell death in the model illustrated in Fig 1A.

One can reduce the complexity of the system by rescaling its variables and parameters without changing the qualitative behaviour. The right column of Table 3 is obtained by dividing the equation for $\dot{P}(t)$ by μP_c, respectively $\dot{D}(t)$ by μD_c, and updating the parameters definitions. In particular, we update $t \leftarrow \mu t$, $P \leftarrow \frac{P}{P_c}$, $D \leftarrow \frac{D}{D_c}$, $Q \leftarrow \frac{D_c}{P_c}$, $k_1 \leftarrow \frac{k_1}{\mu}$, $k_2 \leftarrow \frac{k_2}{\mu}$ and $R \leftarrow \frac{R}{\pi}$. The distribution of the protein content at division is altered analogously. Thus, cells divide if the amount of functional proteins has reached a critical threshold $P(t) = 1$. Subsequently, the cell is considered death when $D(t) = 1$. As a result, the overall parameter space reduces from 7 to 5 dimensions.

model	original	non-dimensionalised
$\dot{\mathbf{P}}(\mathbf{t})$	$\mu P(t)\left(g-\frac{D(t)}{D_c}\right)$<br>$-k_1 P(t)$<br>$+k_2\frac{RD_c}{\pi}\sin\left[\frac{\pi}{R}\frac{D(t)}{D_c}\right]$	$P(t)\,(g-D(t))$<br>$-k_1 P(t)$<br>$+k_2 RQ\sin\left[\frac{D(t)}{R}\right]$
$\dot{\mathbf{D}}(\mathbf{t})$	$+k_1 P(t)$<br>$-k_2\frac{RD_c}{\pi}\sin\left[\frac{\pi}{R}\frac{D(t)}{D_c}\right]$	$+\frac{k_1}{Q}P(t)$<br>$-k_2 R\sin\left[\frac{D(t)}{R}\right]$
**parameter bounds**	$P\in[0,P_c],\quad D\in[0,D_c]$<br>$g\geq 1,\quad \mu,k_1,k_2>0$<br>$R\geq 1$	$P\in[0,1],\quad D\in[0,1]$<br>$g\geq 1,\quad k_1,k_2>0$<br>$R\geq\pi^{-1},\ Q\geq 0$
**cell division**	$P(t)=P_c$	$P(t)=1$
**content at division**		
**in mother :**		
intact P	$sP_c-re(1-s)D$	$s-re(1-s)QD$
damaged D	$sD+re(1-s)D$	$sD+re(1-s)D$
**in daughter :**		
intact P	$(1-s)P_c+re(1-s)D$	$(1-s)+re(1-s)QD$
damaged D	$(1-s)D-re(1-s)D$	$(1-s)D-re(1-s)D$
**cell death**	$D(t)=D_c$	$D(t)=1$
**# parameters**	7	5

Table 3: Comparison between the model before and after non-dimensionalisation.

As an inevitable consequence of this non-dimensionalisation, a new parameter $Q=\frac{D_c}{P_c}$ emerges from the system. Q corresponds to the resilience to damage of the cell, and constitutes an important parameter in single-cell replicative ageing [4]. Large values of Q lead to a long replicative lifespan and can be associated with a large volume increase of the cell during ageing.

References

[1] Broach JR. Nutritional control of growth and development in yeast. Genetics. 2012;192(1):73–105. doi:10.1534/genetics.111.135731.

[2] Lindner AB, Madden R, Demarez A, Stewart EJ, Taddei F. Asymmetric segregation of protein aggregates is associated with cellular aging and rejuve-

nation. Proceedings of the National Academy of Sciences. 2008;105(8):3076–3081. doi:10.1073/pnas.0708931105.

[3] Liu B, Larsson L, Caballero A, Hao X, Öling D, Grantham J, et al. The Polarisome Is Required for Segregation and Retrograde Transport of Protein Aggregates. Cell. 2010;140(2):257–267. doi:10.1016/j.cell.2009.12.031.

[4] Borgqvist J, Welkenhuysen N, Cvijovic M. Synergistic effects of repair, resilience and retention of damage determine the conditions for replicative ageing. Scientific Reports. 2020;10(1):1–15. doi:10.1038/s41598-020-58444-2.

[5] Erjavec N, Cvijovic M, Klipp E, Nystrom T. Selective benefits of damage partitioning in unicellular systems and its effects on aging. Proceedings of the National Academy of Sciences. 2008;105(48):18764–18769. doi:10.1073/pnas.0804550105.

[6] Clegg RJ, Dyson RJ, Kreft JU. Repair rather than segregation of damage is the optimal unicellular aging strategy. BMC Biology. 2014;12(1). doi:10.1186/s12915-014-0052-x.

[7] Nyström T, Liu B. The mystery of aging and rejuvenation-a budding topic. Current Opinion in Microbiology. 2014;18(1):61–67. doi:10.1016/j.mib.2014.02.003.

[8] McClellan AJ, Tam S, Kaganovich D, Frydman J. Protein quality control: Chaperones culling corrupt conformations. Nature Cell Biology. 2005;7(8):736–741. doi:10.1038/ncb0805-736.

[9] Chen B, Retzlaff M, Roos T, Frydman J. Cellular strategies of protein quality control. Cold Spring Harbor Perspectives in Biology. 2011;3(8):1–14. doi:10.1101/cshperspect.a004374.

[10] Vilchez D, Saez I, Dillin A. The role of protein clearance mechanisms in organismal ageing and age-related diseases. Nature Communications. 2014;5:1–13. doi:10.1038/ncomms6659.

[11] Levine RL. Carbonyl modified proteins in cellular regulation, aging, and disease. Free Radical Biology and Medicine. 2002;32(9):790–796. doi:10.1016/S0891-5849(02)00765-7.

[12] Santra M, Dill KA, De Graff AMR. Proteostasis collapse is a driver of cell aging and death. Proceedings of the National Academy of Sciences of the United States of America. 2019;116(44):22173–22178. doi:10.1073/pnas.1906592116.

[13] Ali Q, Dainese R, Cvijovic M. Adaptive damage retention mechanism enables healthier yeast population. Journal of Theoretical Biology. 2019;473:52–66. doi:10.1016/j.jtbi.2019.04.005.

[14] Ginzberg MB, Kafri R, Kirschner M. On being the right (cell) size. Science. 2015;348(6236). doi:10.1126/science.1245075.

[15] Zhou C, Slaughter BD, Unruh JR, Eldakak A, Rubinstein B, Li R. Motility and segregation of Hsp104-associated protein aggregates in budding yeast. Cell. 2011;147(5):1186–1196. doi:10.1016/j.cell.2011.11.002.

[16] Kinkhabwala A, Khmelinskii A, Knop M. Analytical model for macromolecular partitioning during yeast cell division. BMC Biophysics. 2014;7(1):1–10. doi:10.1186/s13628-014-0010-6.

[17] Paoletti C, Quintin S, Matifas A, Charvin G. Kinetics of Formation and Asymmetrical Distribution of Hsp104-Bound Protein Aggregates in Yeast. Biophysical Journal. 2016;110(7):1605–1614. doi:10.1016/j.bpj.2016.02.034.

[18] Andrade-Restrepo M. Is Aggregate-Dependent Yeast Aging Fortuitous? A Model of Damage Segregation and Aggregate Dynamics. Biophysical Journal. 2017;113(11):2464–2476. doi:10.1016/j.bpj.2017.09.033.

[19] Liu B, Larsson L, Franssens V, Hao X, Hill SM, Andersson V, et al. Segregation of protein aggregates involves actin and the polarity machinery. Cell. 2011;147(5):959–961. doi:10.1016/j.cell.2011.11.018.

[20] Spokoini R, Moldavski O, Nahmias Y, England JL, Schuldiner M, Kaganovich D. Confinement to Organelle-Associated Inclusion Structures Mediates Asymmetric Inheritance of Aggregated Protein in Budding Yeast. Cell Reports. 2012;2(4):738–747. doi:10.1016/j.celrep.2012.08.024.

[21] Saarikangas J, Barral Y. Protein aggregates are associated with replicative aging without compromising protein quality control. eLife. 2015;4(e06197):1—-24. doi:10.7554/eLife.06197.

[22] Coelho M, Tolić IM. Asymmetric damage segregation at cell division via protein aggregate fusion and attachment to organelles. BioEssays. 2015;37(7):740–747. doi:10.1002/bies.201400224.

[23] Hill SM, Hanzén S, Nyström T. Restricted access: spatial sequestration of damaged proteins during stress and aging. EMBO reports. 2017;18(3):377–391. doi:10.15252/embr.201643458.

[24] Saarikangas J, Caudron F, Prasad R, Moreno DF, Bolognesi A, Aldea M, et al. Compartmentalization of ER-Bound Chaperone Confines Protein Deposit Formation to the Aging Yeast Cell. Current Biology. 2017;27(6):773–783. doi:10.1016/j.cub.2017.01.069.

[25] Lengefeld J, Barral Y. Asymmetric Segregation of Aged Spindle Pole Bodies During Cell Division: Mechanisms and Relevance Beyond Budding Yeast? BioEssays. 2018;40(8):1–9. doi:10.1002/bies.201800038.

[26] Haynes CM, Titus EA, Cooper AA. Degradation of misfolded proteins prevents ER-derived oxidative stress and cell death. Molecular Cell. 2004;15(5):767–776. doi:10.1016/j.molcel.2004.08.025.

[27] Moreno DF, Aldea M. Proteostatic stress as a nodal hallmark of replicative aging. Experimental Cell Research. 2020;394(2):1–14. doi:10.1016/j.yexcr.2020.112163.
