# Supplementary material for: The synergy of damage repair and retention promotes rejuvenation and prolongs healthy lifespans in cell lineages
Source: PLoS Comput Biol. 2020 Oct 12;16(10):e1008314. doi: 10.1371/journal.pcbi.1008314 (PMC7598927; doi:10.1371/journal.pcbi.1008314)
Supplement: S2 Text — Detailed description and justification of the repair term with special focus on the repair capacity R. (PDF) [file pcbi.1008314.s010.pdf]

The synergy of damage repair and retention promotes rejuvenation and prolongs healthy lifespans in cell lineages

S2 Text : Repair term with the repair capacity R

Barbara Schnitzer [1], Johannes Borgqvist [1], Marija Cvijovic [1*]

[1] Department of Mathematical Sciences, University of Gothenburg, Sweden
[*] corresponding author: Marija Cvijovic, marija.cvijovic@chalmers.se

Cells have an extensive protein quality control system to transform back damaged to functional proteins by refolding or degradation and new synthesis by so-called chaperones and the ubiquitin-proteasome system [2–4]. We assume that this happens with a repair rate k_2. Since however also the protein quality control system suffers from too much damage [5,6], we chose a non-linear term for repair dependent on how close the cell is to cell death. This effect is reflected in the repair capacity R that sets how much the effective repair declines with age.

Repair in the non-dimensionalised single-cell ODE model is modelled by

$$r(D) = k_2 R \sin\left[\frac{D(t)}{R}\right].$$

The repair capacity is incorporated by a scaled sine function. Only maximally the first half of a period is captured by the model due to the bounds on $P(t) \in [0,1]$, $D(t) \in [0,1]$ and $R \in [\pi^{-1}, \infty]$. R changes the period length of the sine and so tunes how much the repair declines with increasing D. As R approaches infinity $\sin\left[\frac{D(t)}{R}\right] \approx \frac{D(t)}{R}$ and the repair velocity becomes a linear function as in [1]. It is equivalent to a very large period length of the sine function. Decreasing R will continuously decrease the period length and eventually when $R = \pi^{-1}$ the repair will decline all the way to 0 again at the cell's maximal damage level $D(t) = 1$. Only in this extreme case exactly half a period of the sine is captured, in all other cases it is less.

In that way, we include two parameters k_2 and R that further have a distinct biological meaning. Since the sine function is linear for small arguments, the behaviour for young cells with small amounts of damage ($D \to 0$) is the same for all values of R. In particular, the rate of repair in that regime corresponds

to k_2. In other words, independent of R cells can repair damage at the same rate k_2 if the damage levels are still low. We denote unlimited repair capacity the case where cells can continue repairing at that same rate throughout the whole life ($R \to \infty$). The efficiency of the repair machinery is not influenced by how old and damaged the cell is. However, the profiles differ increasingly during ageing for decreasing R (Fig 2). The lower the capacity to repair is, the more affected is the repair machinery with increasing damage levels in the cell and the effective repair rate drops towards old age, which is likely a more biologically realistic scenario. The other extreme would then correspond to when cells cannot repair at all anymore when close to death, denoted as decline in repair capacity ($R = \pi^{-1}$). Values for R in the valid range $R \in [\pi^{-1}, \infty]$ allows modelling even all scenarios in between the two described extremes, as for example a saturated repair capacity in which cells reach a maximal effective repair rate that is then maintained during ageing, similar to Michaelis-Menten kinetics.

Even though the repair term in our model is not derived from first principles and does not explain mechanistic details of the repair machinery, it helps to understand the consequences of a specific profile, independent of how cells can biologically reach that scenario. Instead of creating several models, there are two distinct biological parameters k_2 and R that can tune various scenarios, which compresses and simplifies the analysis and comparison of the model.

References

[1] Borgqvist J, Welkenhuysen N, Cvijovic M. Synergistic effects of repair, resilience and retention of damage determine the conditions for replicative ageing. Scientific Reports. 2020;10(1):1–15. doi:10.1038/s41598-020-58444-2.

[2] McClellan AJ, Tam S, Kaganovich D, Frydman J. Protein quality control: Chaperones culling corrupt conformations. Nature Cell Biology. 2005;7(8):736–741. doi:10.1038/ncb0805-736.

[3] Chen B, Retzlaff M, Roos T, Frydman J. Cellular strategies of protein quality control. Cold Spring Harbor Perspectives in Biology. 2011;3(8):1–14. doi:10.1101/cshperspect.a004374.

[4] Vilchez D, Saez I, Dillin A. The role of protein clearance mechanisms in organismal ageing and age-related diseases. Nature Communications. 2014;5:1–13. doi:10.1038/ncomms6659.

[5] Levine RL. Carbonyl modified proteins in cellular regulation, aging, and disease. Free Radical Biology and Medicine. 2002;32(9):790–796. doi:10.1016/S0891-5849(02)00765-7.

[6] Santra M, Dill KA, De Graff AMR. Proteostasis collapse is a driver of cell aging and death. Proceedings of the National Academy of Sciences of the United States of America. 2019;116(44):22173–22178. doi:10.1073/pnas.1906592116.
