# Supplementary material for: The synergy of damage repair and retention promotes rejuvenation and prolongs healthy lifespans in cell lineages
Source: PLoS Comput Biol. 2020 Oct 12;16(10):e1008314. doi: 10.1371/journal.pcbi.1008314 (PMC7598927; doi:10.1371/journal.pcbi.1008314)
Supplement: S3 Text — Parameter choices and pseudo-code for relevant computational procedures. (PDF) [file pcbi.1008314.s011.pdf]

The synergy of damage repair and retention promotes rejuvenation and prolongs healthy lifespans in cell lineages

S3 Text : Computational guidelines

Barbara Schnitzer [1], Johannes Borgqvist [1], Marija Cvijovic [1*]

[1] Department of Mathematical Sciences, University of Gothenburg, Sweden
[*] corresponding author: Marija Cvijovic, marija.cvijovic@chalmers.se

1 Fixed parameters and conventions

name	parameter	value	ref.
size proportion	s	0.6370	[1]
damage resilience	Q	2.5526	[1]
growth factor	g	1.05	[1]
random effects in rate parameters	σ	0.005	[1]
no retention<br>$\hat{=}$ damage in mother at division	re	0.0<br>63.70%	this study
retention<br>$\hat{=}$ damage in mother at division	re	0.2957<br>74.43%	this study, [1]
unlimited repair capacity	R	10^3	this study
decline in repair capacity	R	π^{-1}	this study
maximal generation in lineage		3	this study
critical threshold defining the health span	D_c	0.5	this study
threshold for healthy cells	h_c	0.3	this study

Table 1: Fixed parameters and conventions used in the paper.

Note that the value of R for unlimited repair is an approximation for $R \to \infty$. P and D are bounded by 1 and within that regime the deviations are neglectable.

2 Create lineage

In most simulations, we generate lineages up to 3 generations. The ODEs of a cell are not coupled to any other cell. Therefore, each cell can be solved individ-

ually. In case there is some coupling between the cells, e.g. time or shared food resources, all ODEs of alive cells have to be solved at the same time instead and dead cells have to be removed from the system.

Algorithm 1 Create lineage (uncoupled cells)

```
Define all relevant parameters
Initialise n founder cell(s) with identifiers 1, ..., n
Set (dynamic) lineage size N ← n
Set maximal generation number M
for i ∈ 1..N do
    while true do
        Solve ODE of cell i until it divides (P(t) = 1) or dies (D(t) = 1)
        Keep track of all wanted properties
        if P(t) = 1 then
            Reset protein content of mother cell according to division rules
        else if D(t) = 1 then
            break
        end if
    end while
    if daughters are in generation m < M then
        Add rls new daughter cells to the lineage according to division rules with
        identifiers N + 1, ..., N + rls
        N ← N + rls
    end if
end for
Save all wanted information
Analyse population properties
```

3 Finding *wildtype* cells in the parameter space

In order to find parameter combinations in the k_1, k_2 and re space that lead to 24 divisions we use an iterative process with adaptive step size. Typically, we fix two of the three dimensions to a value and find the third one. Algorithm 2 shows an example for adapting k_2 if all other parameters are set.

Algorithm 2 Finding *wildtype* cells

Define all relevant parameters
Set initial conditions of cell to $P(0) = 1 - s$, $D(0) = 0$
Set wanted replicative lifespan $rls^* = 24$
Set initial value of the parameter to adapt $k_2 = 0$
Set initial step size Δk_2 and minimal step size $\Delta k_2 \geq \delta$

Coarse search :
while true **do**
    $k_2 \leftarrow k_2 + \Delta k_2$
    Run single-cell model
    if $(rls > rls^*)$ **then**
        break
    end if
end while

Fine search with adaptive step size :
while true **do**
    $\Delta k_2 \leftarrow \Delta k_2 \cdot 0.5$
    Run single-cell model
    if $(rls == rls^*$ or $\Delta k_2 < \delta)$ **then**
        break
    else if $rls > rls^*$ **then**
        $k_2 \leftarrow k_2 - \Delta k_2$
    else
        $k_2 \leftarrow k_2 + \Delta k_2$
    end if
end while

wildtype is found if $rls == rls^*$ and the corresponding parameter is k_2

In the same manner, one can start with many cells and allow for parameter variations in k_1 and k_2 according to non-linear mixed effects (equation (2) in the paper) and check if the average value of the respective parameter is around the wanted replicative lifespan with some tolerance. The algorithm does not necessarily have to be 24 as it is for the *wildtype* yeast cells, but works for any preset replicative lifespan. Note that when varying the retention factor, it is computationally more efficient to start from a high value and reduce it stepwise. Corresponding signs have to be adapted.

In the paper, we often compare four cases, which correspond to following

parameter combinations. We chose to fix the damage formation rate $k_1 = 0.4$ and adapt the repair rate correspondingly (see also S1 Fig).

**repair mechanism**	**retention**	$(\mathbf{re}, \overline{\mathbf{k}}_\mathbf{1}, \overline{\mathbf{k}}_\mathbf{2}, \mathbf{R})$
unlimited repair capacity	retention	(0.2957, 0.4, 0.09219, 10^3)
	no retention	(0.0, 0.4, 0.02438, 10^3)
decline in repair capacity	retention	(0.2957, 0.4, 0.13750, π^{-1})
	no retention	(0.0, 0.4, 0.03125, π^{-1})

Table 2: Parameter combinations for *wildtype* populations.

The range of values of k_1 and k_2 (Fig 2) generating wild-type cells is in agreement with previous computational and experimental work [2–4] underlining the validity of the conclusions. However, often the estimation relies on various assumptions. Most importantly, experiments usually contain one specific type of damage such that we have to assume that the rate is similar even for other types. Further parameters are sometimes estimated indirectly and we have to assume that the underlying dependency is linear, which is not necessarily true. Clegg and colleagues implemented a similar damage formation term using values in the same range as we do [2]. The response of chaperones to damage formation measured by Saarikagas and colleagues ($\approx 0.1\frac{1}{h}$), can be interpreted as the increase of the damage while the clearance rate of protein deposits ($\approx 0.07 - 0.15\frac{1}{h}$) as the repair rate [4]. Further, the estimated aggregation formation rate by *Paoletti et al* ($\approx 0.21\frac{1}{h}$) can be interpreted as the damage formation rate [3]. In *E.Coli*, another organism often used in ageing studies, a value of $\approx 0.72\frac{1}{h}$ for the rate of protein misfolding has been obtained [5].

Note that k_1 and k_2 in our model are non-dimensionalised, such that these values have to be multiplied by the growth rate μ ($\approx 0.5\frac{1}{h}$, estimated in [6] assuming full availability of resources) to go back to full dimension and to be compared with experimental values. Consequently, we can justify that the order of the values in our model is realistic.

4 Average initial conditions in a cell lineage

For all population studies the founder cells start with initial conditions $\overline{P}(0)$ and $\overline{D}(0)$ which are specific to a set of parameters. Starting with an average cell gives rise to more realistic populations and facilitates the analysis since it is not necessary to simulate a large number of generations to get a representative view of the populations. The initial conditions are found according to algorithm 3. The algorithm converges independent of the age of the founder cell up to a

certain precision. With $\epsilon = 10^{-3}$ no numerical issues were faced and the values are sufficiently precise for our purpose.

Algorithm 3 Finding average initial conditions

Define all relevant parameters
Set initial conditions of founder cell to $P(0) = 1 - s$, $D(0) = 0$
Set tolerance ϵ

while true **do**
  Create founder cell with $P(0)$ and $D(0)$
  Run population model and obtain lineage
  $\overline{P}(0) \leftarrow$ average intact protein content at birth in lineage
  $\overline{D}(0) \leftarrow$ average damaged protein content at birth in lineage
  if ($|\overline{P}(0) - P(0)| \leq \epsilon$ and $|\overline{D}(0) - D(0)| \leq \epsilon$) **then**
    break
  else
    $P(0) \leftarrow \overline{P}(0)$
    $D(0) \leftarrow \overline{D}(0)$
  end if
end while

$\overline{P}(0)$ and $\overline{D}(0)$ are the average initial conditions

References

[1] Borgqvist J, Welkenhuysen N, Cvijovic M. Synergistic effects of repair, resilience and retention of damage determine the conditions for replicative ageing. Scientific Reports. 2020;10(1):1–15. doi:10.1038/s41598-020-58444-2.

[2] Clegg RJ, Dyson RJ, Kreft JU. Repair rather than segregation of damage is the optimal unicellular aging strategy. BMC Biology. 2014;12(1). doi:10.1186/s12915-014-0052-x.

[3] Paoletti C, Quintin S, Matifas A, Charvin G. Kinetics of Formation and Asymmetrical Distribution of Hsp104-Bound Protein Aggregates in Yeast. Biophysical Journal. 2016;110(7):1605–1614. doi:10.1016/j.bpj.2016.02.034.

[4] Saarikangas J, Barral Y. Protein aggregates are associated with replicative aging without compromising protein quality control. eLife. 2015;4(e06197):1—-24. doi:10.7554/eLife.06197.

[5] Santra M, Dill KA, de Graff AMR. How Do Chaperones Protect a Cell's Proteins from Oxidative Damage? Cell Systems. 2018;6(6):743–751.e3. doi:10.1016/j.cels.2018.05.001.

[6] Snoep JL, Mrwebi M, Schuurmans JM, Rohwer JM, Teixeira de Mattor MJT. Control of specific growth rate in Saccharomyces cerevisiae. Microbiology. 2009;155(5):1699–1707. doi:10.1099/mic.0.023119-0.
