# Supplementary material for: The synergy of damage repair and retention promotes rejuvenation and prolongs healthy lifespans in cell lineages
Source: PLoS Comput Biol. 2020 Oct 12;16(10):e1008314. doi: 10.1371/journal.pcbi.1008314 (PMC7598927; doi:10.1371/journal.pcbi.1008314)
Supplement: Attachment — Submitted filename: Plos Rejuvenation rebuttal_final.pdf [file pcbi.1008314.s012.pdf]

Comments to the Authors:
Please note here if the review is uploaded as an attachment.

Reviewer #1: The paper developed a dynamical model for damage accumulation in budding yeast cells including the production of damaged proteins and the asymmetric segregation of proteins at division quantified by the retention factor. If I understand correctly, the paper's main conclusion is that damage retention and early investment in repair are beneficial for healthy ageing in yeast cell populations.

We would like to thank the reviewer for a careful and thorough evaluation of the manuscript. We have received much appreciated constructive and valuable comments and suggestions that we addressed below.

My main concern is that the dynamical model appears to be very specific, especially in terms of the mathematical form of the repair capacity. It appears that many of the conclusions of this paper relies on the particular forms they choose and the generality of their results are not justified.

We would like to thank the reviewer for pointing this out, and we do admit that we were less careful when defining the main scope and aim of the proposed model. The dynamical model presented in this work aims at describing replicative ageing phenomena in yeast. We first start with a comprehensive description of replicative ageing on a single-cell level, and we extend the model, by implementing a non-linear mixed effect approach to model replicative aging of the whole yeast population. Thus, the specificity comes in the context of the organism (asymmetrically dividing budding yeast), the type of ageing - replicative ageing characterised by accumulation of age-related damage.

With the proposed modelling framework, we aimed to investigate the most beneficial repair mechanism in the context of replicative ageing in yeast, so that the conclusions indeed rely on the repair capacity. Further, we also investigated how the combination of retention and repair (in particular the repair capacity) alters how cells age and how that affects the overall population.

From the current experimental evidences, it is not completely understood how the repair machinery is affected by ageing and which consequences it has on rejuvenation and health span. By incorporating several proposed repair mechanisms (continued, saturated and declining) within one function we can easily compare them on both individual and population level as well as their effect on properties like rejuvenation and health span.

Our modelling approach describing replicative ageing of budding yeast on both individual and population level permits this type of study. Further, the model is easily adaptable to test similar effects in symmetrically dividing systems as well, thus it can be also used to test evolutionary hypothesis to elucidate the role of damage retention and repair in the context of disposable soma theory.

Moreover, the paper is not clearly written and some of the formulas and results are not explained. Below are some more minor comments.

We have now revised several sections of the ms to improve the readability. We, also, include additional tables in the supplementary section S1: Table S1 summarises and motivates the continues part of the model (dynamics of intact and damaged proteins) and Table S2 describes discrete parts of the model – cell division and cell death. Further we include a type of numerical sensitivity analysis in supplementary section S4 to justify the parameter choice and show generality of the qualitative results.

(1) It is unclear what is "r" in Eq.1 and it is also unclear why the authors choose sin function which can lead to negative value.

r(D) represents the repair term. A clarifying sentence for r is now included in the revised ms (see Ln 101). The advantage of describing the repair with a sine function is that we could explain and consequently compare different repair mechanisms that are still elusive from experimental investigations.

Negative values for the sine function do not occur, as we maximally take into account half a period (for details see Supplementary sections S1 and S2). Along with Eq 1 we included parameter bounds that also imply the half a period of a sine function.

The sine function becomes linear for small arguments. In that way we can incorporate all profiles from linear over saturated to declining steered by two distinct parameters: the repair rate k2 which determines the slope in the beginning, and the repair capacity which alone steers if the behaviour of the repair towards the end of the cell's life. If we had instead used several model structures, e.g. by a linear term k2*D, a non-linear term k2 * D (R - D) or k2 * D /(R + D), we would not get this clear separation and in our opinion it would be more difficult to compare the profiles in the way we aimed to do it.

(2) The right panel of Fig.1A is unclear, for example, the meaning of black and green arrows are not explained.

The arrows aimed to represent 'movement' of damage (black) and intact (green) components from mother to daughter (and vice versa). We now extended the description in the caption of Fig. 1A and referred more clearly to the formulae explanations in the supplementary section S1.

(3) The meaning of the resilience to damage Q is not explained but it looks quite important as it appears both in the dynamics of protein level and the retention of protein at cell division.

Resilience to damage Q is defined as a quotient between death (D_{death} and division (P_{div}) thresholds ($Q = D_{death} / P_{div}$) and can be interpreted as the capacity of the cell to cope with damage. A high value of Q corresponds to an organism that is resilient to damage with a long replicative life span. The parameter Q was the focus of our previous work (Borgqvist et al., 2020 Scientific Reports) where it was also estimated using time-lapse microscopy imaging of dividing young and old yeast cells that were previously grown in a microfluidics device enabling continued availability of nutrients required for cell growth and division.

In SI Table S4 we provide values and source for each parameter. We also added another clarification sentence in the main text (see Ln 97-99).

(4) In the section of population model, it's not clear what is the "explicit knowledge about all mother-daughter relations". It looks like k1 and k2 are drawn independently between mother and daughter celle.

By "explicit knowledge about all mother-daughter relations" we mean genealogical history of each cell, i.e. for each daughter cell it is exactly known from which mother and consequently grandmother it is derived from. In budding yeast, as the mother is ageing, the first daughters she produces are born damage-free, however as the mother is getting older the new-born daughters start inheriting damage as well and are thus considered to be prematurely old. By tracing each cell, we could define a 'lineage position' to capture this inheritance and, we could deduce the effect of, in some sense 'birth order', on properties like rejuvenation and health span.

When it comes to the rate parameters, indeed, there is no connection between mother and daughter. We clarified this in revised version (see Ln 139-140).

(5) Line 149-154 on two distinct cases of the repair capacity are unclear.

We have re-organized the paragraph by adding additional explanation and moving it further up where we explain the model (see Ln 106-117).

In brief, two distinct cases of the repair capacity considered in this work are: (1) unlimited repair capacity, corresponding to a constant rate of repair throughout the cell's life independent of the damage levels in the cell, and (2) decline in repair capacity, corresponding to a continuous effect of damaged components on the repair machinery, especially evident towards the end of the cell's life where the effective rate of repair drops to zero.

(6) Line 196-198 on the discussion of Figure 3B argues that the damage at birth in daughters is lower when there is retention. The authors may want to show the results with retention and without retention in the same figure to be more clear.

We added an additional figure in the supplementary (Figure S7) that shows only the damage in the daughter for all four cases and referred to it in the text (see Ln 210 in revised manuscript and in the caption of Figure 3B).

(7) On line 184, it is unclear how the population size is defined as it changes with time. I guess the authors are computing the population size after a constant time starting from the same initial condition.

We define population size in Box 2 (Ln 151), i.e. we count the number of cells in the lineage up to generation 3. In Figure 3C we also investigate the number of alive cells at a certain time point, which could also be termed population size, however deliberately we do not call it population size to avoid confuse, since the latter is rather an indication of the growth rate of the population in the exponential phase.

(8) It is unclear how unlimited repair capacity leads to increased generation time.

Cells with unlimited repair capacity accumulate damage faster (Fig 3B) and can keep high damage levels for a longer time because the repair works with a constant rate throughout the life. Since damage also acts on growth these cells typically cannot grow as fast to reach the critical size that allows division. In that way, even though initially counterintuitive, unlimited repair capacity actually leads to longer generation times. We added additional clarification in the section "Decline in repair capacity results in lower damage levels and prolonged health spans by shortening generation time" (see Ln 248-254).

Reviewer #2: Schnitzer et al. developed a dynamic model of replicative ageing in studying damage accumulation in a single cell. Through the modeling approach the authors showed that active damage retention lowers damage levels in the population by reducing the variability across the lineage. They also found that repairing damage efficiently in early life as opposed to intervention in the later stage could slow ageing progress caused by damage retention. The metric "health span" investigated in this study is also interesting which could be used to quantify topics related to healthy ageing and quality of life.

We would like to thank the reviewer for a careful and thorough evaluation of the manuscript. We have received much appreciated constructive and valuable comments and suggestions that we addressed below.

My main concern is about the generality of the modeling results. More specifically, the conclusions of this study were mainly made based on one single set of parameter values shown in Table S2. The authors should provide justifications about how those parameter values are chosen. For parameter values without experimental/literature backup, the authors should also investigate and report how the results and conclusions would be impacted/altered by conducting parameter sensitivity analysis.

The model and parameters in this work are chosen in such a way that they describe replicative ageing in budding yeast, thus, certain parameters needed to be constrained to only single parameter values as they define wildtype (wt) yeast cell, i.e. cells with the given replicative lifespan (of 24) describing a virgin daughter with no damage (Fig 2). This was the basis of the analysis.

Key parameters defining wt cells are the rate of damage production (k1) and the rate of repair (k2). We now included population simulations in SI S4, where, in brief, we show that the choice of k1 does not change the qualitative results. We chose k1=0.4 since it is one of the smallest values where no retention and retention can be compared. Furthermore, larger k1 leads to longer generation times and thus lifetimes of the cells resulting in unreasonable computation times. From those choices, k2 is automatically determined given re and R and the parameter grid of wildtype cells.

Retention of damage (re) can take range of values, where minimal value of 0 means that the damage segregation is only size dependent and maximal theoretically derived value is 0.2957.

In the course of this work, we compare the cells with and without damage retention to elucidate its effect on individual cell and on the whole population in respect to rejuvenation and health span.

Repair capacity (R) can also take range of values, where 10^3 represents unlimited repair capacity and π^{-1} represents the decline in repair capacity. By describing the repair with the sine function, we could explain and consequently compare different repair mechanisms that are still elusive from experimental investigations. For the analysis in this work we simulate only those two extreme values since everything in between behaves smooth and monotonous.

Resilience to damage Q is defined as a quotient between death (D_c) and division (P_c) thresholds ($Q = D_c / P_c$) and can be interpreted as the capacity of the cell to cope with damage. A high value of Q corresponds to an organism that is resilient to damage with a long replicative life span. The parameter Q was the focus of our previous work (Borgqvist et al., 2020 Scientific Reports) where it was also estimated using time-lapse microscopy imaging of dividing young and old yeast cells that were previously grown in a microfluidics device enabling continued availability of nutrient required for cell growth and division.

To reflect this, we have rewritten several section of the ms (in red in the revised version) and have now included additional Tables in the Supplementary Table S1 and S2.

In Fig. 1A, authors should address the rational behind the first term $P(t)(g - D(t))$, and why there is no rate constant associated with this term.

The term $P(t)(g - D(t))$, represents growth and is given in its non-dimensionalised form and therefore the rate constant is not present. In the Supplementary we presented model in both original and non-dimensionalised form (Table S3).

The growth rate in its ordinary form has a rate constant associated with this term, and is presented as $\mu P(t)(g-D(t)/D_c)$, where μ is a growth rate, which is constant as it is assumed that an abundance of substrate is available for each cell, which typically occurs in a microfluidics system with continuous inflow of nutrient rich media. As the rate of cell growth declines with increasing amounts of damage, the unit-less factor $(g-D(t)/D_c)$ is included in the growth term. The parameter g is a positive number that is larger than 1 and determines the decline in growth rate. Thus, as D approaches the death threshold D_c the difference $(g-D(t)/D_c)$ will decrease corresponding to a slower growth rate.

Minor point, in Line 301, "generation time of each cell to it size increase", it looks like there is a typo here. The authors may want to write "its" instead of "it".

This is now corrected in the text.

Have all data underlying the figures and results presented in the manuscript been provided?
Large-scale datasets should be made available via a public repository as described in the *PLOS Computational Biology* data availability policy, and numerical data that underlies graphs or summary statistics should be provided in spreadsheet form as supporting information.

Reviewer #1: None

Reviewer #2: Yes

Data and code are available on the github repository (see Ln 415). We added an additional folder called PublicationData that includes data underlying each figure in the manuscript.
