# Supplementary material for: The synergy of damage repair and retention promotes rejuvenation and prolongs healthy lifespans in cell lineages
Source: PLoS Comput Biol. 2020 Oct 12;16(10):e1008314. doi: 10.1371/journal.pcbi.1008314 (PMC7598927; doi:10.1371/journal.pcbi.1008314)
Supplement: Attachment — Submitted filename: Rebuttal.pdf [file pcbi.1008314.s013.pdf]

Reviewer 3: Summary:

Schnitzer et al. introduce a dynamical model which helps study the effect of intracellular processes at a population level. They also define interesting new measures as the rejuvenation index and the health span that help analyze some of the characteristics of budding yeast cells at a population level. They find evidence of a trade-off between damage repair and damage retention to replicate replicative life-spans in WT cells. They find that increased retention leads to larger, healthier populations and decreases the variability of the replicative life-span of offspring. Moreover they find that a decline in repair capacity reduces damage and improves health in contrast to unlimited (constant) repair capacity.

General Comments:

The main interest of the paper; using mathematical and computational analysis to understand how intracellular processes affect observables at a population level is innovative and interesting and has not yet been put to use thoroughly in existing bibliography (to my knowledge). The potential to this approach is vast and can have very strong implications as it provides new ways of analyzing feasible experimental data and its relation to intracellular processes which are difficult to study or analyze directly. The first main result, that active damage retention lowers damage levels, is not very striking and has been discussed in a fair amount of papers in the past ten to twenty years. The second main result, that repairing damage in early life can be more effective than continuously and constantly throughout life, is more captivating as it links to existing theories of ageing (the disposable soma theory) and to existing experimental results. The introduction of new measures at a population level is very promising and can help reduce the gap between existing theories of intracellular processes and experimental observables.

Although the methods and some of the results can be of interest in the community, I agree with the previous referees that the model seems too specific and that the conclusions rely strongly on the functional forms chosen (more details on this below). Furthermore, comparisons or referrals to available published experimental work are very limited. Improving this aspect could have important consequences in terms of validating the simulations' results and reducing the degrees of freedom in the models' dimensional space. Moreover, I believe that the use of the term “active” when referring to retention can be misleading (see detailed comment below) and that the equations used to describe intracellular processes remain unjustified and are not necessarily in line with the existing mathematical theory, which decreases the impact of the paper's discoveries.

We would like to thank the reviewer for a very careful and thorough evaluation of the manuscript. We have received very much appreciated, constructive and valuable comments and suggestions. We carefully addressed all comments from the reviewer which lead to a substantial revision of the manuscript, in particular the supplementary information.

We would like to emphasise in the beginning that the population model presented in this work builds on our previously published comprehensive dynamical model of yeast replicative ageing that was validated using time-lapse microscopy imaging of dividing young and old yeast cells that were previously grown in a microfluidics device enabling continued availability of nutrients required for cell growth and division (Borgqvist et al., Scientific Reports (2020)). In that, as well as in this work, we aimed at constructing models in a so called modular approach, where we take critical ageing components (that are relevant not only for yeast, but for other organism as well) and represent them as modules. This approach, of course has both advantages and disadvantages. To capture the complex process of ageing, in some instances modules are very general. The generality comes to a large extend from the fact that the level of detail of these modules is low, and various molecular mechanisms are combined in a single parameter, e.g. for damage formation, repair and retention. At the same time, available experimental data are rather very specific, in general focusing on one very specific mechanism or for example measures of damage of single proteins. However, the modular approach has been successfully applied in many available replicative ageing models (Chao et al. PLOS Genetics (2010), Clegg et al., BMC Biology (2014), Song et al., BMC Bioinformatics (2019), Borgqvist et al., Scientific Reports (2020), Erjavec et al., PNAS (2008), ...) that could typically at least be partly experimentally verified.

A clear advantage is, as the reviewer also already noted, and what is the aim of this work is to go a step further and use these types of models to infer population properties that are not elusive from experiments yet, and provide useful measures that can guide experiments in the future and even further look at ageing from a more holistic view by laying a ground work for detailed large scale kinetic models describing ageing.

In the following, we address all comments in more detail.

Major points:

1. The equations used for the intracellular dynamics in the model are still not fully justified. In particular the equations concerning the distribution of damage after cell division. These equations don't seem consistent with more explicit

existing models of aggregate dynamics and with the theory of escape times of diffusing particles inside confined domains (see work from Holcman and Schuss). Also, are these equations based on a 2 or a 3 dimensional representation of the cell? It should be at least explained why this is different or how it relates to them (if it improves them, focuses on something else etc.). See for instance: A. Kinkhabwala, A. Khmelinskii, and M. Knop, BMC biophysics 7, 10 (2014), M. Andrade-Restrepo, Biophysical journal 113, 2464 (2017), C. Paoletti, S. Quintin, A. Matifas, and G. Charvin, Biophysical journal 110, 1605 (2016). Moreover, and as explained below, significant retention can occur as well due to passive effects, hence claiming that the retention term in the equation is only a consequence of active processes is not necessarily true.

The explanation for the sine in the damage repair term given to the previous reviewer should be added to the paper. Otherwise, it seems artificial. More minor comments on these equations are below.

To improve the explanation and justification of all respective model features, we revised in depth the model description in the supplementary (SI S1). In particular, regarding the repair term we also included an updated explanation in the manuscript (l. 109-127) and the supplementary (SI S2).

The cell division process implemented in our model was previously described in Erjavec et al., PNAS (2008), Clegg et al., BMC Biology (2014), Ali et al., Journal of Theoretical Biology (2019) and Borgqvist et al., Scientific Reports (2020). We use the number of functional (P) and damaged (D) proteins, and approximate the cell size by their sum. Hence, we do not include spatial dimensions, but only treat each cell as a pool of functional and damaged proteins. This pool is distributed between daughter and mother at cell division by firstly ensuring mass conservation, i.e. the total amount of proteins does not change, and secondly steering the asymmetry in the concentration of damaged proteins between mother and daughter, i.e. how many proteins are retained in the mother.

We believe that our model is not contradictory to previous mathematical and computational models, including the ones mentioned above, however we have a different focus and level of detail. The most important difference is that in our model we do not distinguish between active and passive retention mechanisms, such that the retention factor re comprises all possible (known and unknown, active and inactive) retention mechanisms. Instead, we only focus on the fact that it leads to an increase in the degree of damage concentration asymmetry between mother and daughter cell. We do not include mechanistic details on the single-cell level of how the cell reaches the asymmetry. In other words, our model zooms out and decreases the level of detail. When damage retention is not present ($re = 0$, no retention) we therefore assume that all functional and damaged proteins are

similar in size and moreover sufficiently small such that the equilibrium between mother and bud is reached fast on the timescale of a cell division. This implies that at separation $s(P+D)$ proteins would be in the mother and $(1-s)(P+D)$ in the daughter, in accordance with the papers mentioned above (Kinkhabwala et al., BMC Biophysics (2014), Andrade-Restrepo, Biophysical Journal (2017), Paoletti et al., Biophyiscal Journal (2016), and e.g. Holcman and Schuss, Springer (2015)). The damage concentration in both compartments is the same (not the absolute number since the compartments are not equally big for budding yeast). By increasing re we switch on all possible retention mechanisms and in that way increase the damage concentration in the mother cell compartment while keeping the size proportion constant. This can be partly caused by aggregation formation that automatically leads to slower diffusion and therefore asymmetry at division since equilibrium is not yet reached. But it can also be caused by more active processes like spatial sequestering or active transport. Since the objective of the project was to investigate the effect of retention on the cell population, we believe that it is not necessary to distinguish between the underlying processes. However, with a different focus, it would definitely be interesting to unravel retention, possibly including spatial dimensions, in our setting as well.

2. As anticipated above, the use of the term "active" is misleading in the paper. While the degree of retention is certainly varied throughout the paper and it can have a strong impact on the results, it is never specified to what degree this retention occurs due to active processes (see previous point as well). Passive processes (confined diffusion, crowded environment, aggregate fusion etc.) can also justify much of the retention observed in real life cells even if the presence of Active-quality-control seems more and more likely. Therefore, while retention is undoubtedly important, the effect of active processes can or can not be significant in the observed results and has not yet been properly analyzed to be able to claim that active retention and not just retention is behind the paper's discoveries. Again, since there is no reference to more explicit and detailed models of intracellular dynamics it seems difficult to justify why retention in the model must be active.

We admit that we were not careful enough with the terminology regarding retention and misused the term active retention from a biological perspective. As already explained in the previous point, our model is too coarse or zoomed out to even distinguish between active and passive retention mechanisms. For that reason we abolished 'active' every time we used it for describing retention in our model. The way retention is implemented in our model it comprises both active and passive retention mechanisms altogether. In other words it is treated as a module (governed by the retention factor re) that introduces damage con-

centration asymmetry between the mother and daughter, independent of how it is actually generated biologically. We justify that by the fact that we are mainly interested in the consequences of increasing damage asymmetry on the population level, not the underlying mechanisms on the single-cell level. We now, in the introduction include what retention can be biologically (l. 43-52) but also what retention is in our model and how it is justified in the model description (l. 131-133 and SI S1). However, it would certainly be interesting to expand retention in our model to incorporate the different levels of detail of retention, on both the single-cell and the population level.

3. Comparisons to available experimental data are very limited. I believe some of the results could be compared with experimental papers concerning replicative life-spans of budding yeast cells and damage accumulation (proportion of damage in mother and daughter cells) and of 2nd-degree rejuvenation. As one reviewer pointed out before, experimental validation or comparisons with experimental data of parameter values could improve the feeling that the equations used are too "artificial" and that results strongly depend on their form. For instance, a decline in repair capacity yields a higher k_2 (Fig. 2) which could alone justify the lower damage levels observed. So the effect would not come from a better strategy but form a higher maximal repair rate.

We do not directly work with specific proteins but with the pool of all proteins such that the level of detail of our model is low. Similarly, we treat retention and repair as a whole without distinguishing between individual mechanisms. Therefore, it is non-trivial to find isolated and directly measurable parameters that can be estimated in a classical sense. We explored the whole parameter space of k_1 and k_2 to find out which values can in principle lead to wildtype yeast cells with a replicative lifespan of around 24 divisions. This already restricts the values of the rate parameters a lot, and only a small subspace of parameter values fulfils this requirement (Fig 2). We have now compared the values reported in the literature to underline the validity of the conclusions, and confirmed that the ranges of k_1 and k_2 in our model are in the agreement with previous computational and experimental work. It is relevant to emphasise, that often the estimation relies on various assumptions. Most importantly, experiments usually contain one specific type of damage such that we have to assume that the rate is similar even for other types. Further parameters are sometimes estimated indirectly and we have to assume that the underlying dependency is linear, which is not necessarily true. We found that Clegg et al., BMC Biology (2014), with a similar damage formation term used values in the same range. Saarinkangas et al., eLife (2015), measured the response of a chaperone to damage formation. Assuming that the increase of this protein is similar to the increase of the damage, we can estimate

the damage formation rate to be $\approx 0.1\frac{1}{h}$. Santra et al., Cell Systems (2018), used $\approx 0.72\frac{1}{h}$ for the rate of protein misfolding, however in E.Coli. Paoletti et al., Biophysical Journal (2016), estimated the aggregation formation rate as $\approx 0.21\frac{1}{h}$ which could be similar to the damage formation rate. Further we can estimate the repair rate by the clearance rate of protein deposits in Saarinkangas et al., eLife (2015), who estimated values between $\approx$ 0.07 and $0.15\frac{1}{h}$. Note that k_1 and k_2 in our model are non-dimensionalised, such that these values have to be multiplied by the growth rate μ ($\approx 0.5\frac{1}{h}$, estimated in Snoep et al., Microbiology (2009), assuming full availability of resources) to go back to full dimension to be compared with experimental values. In that way we can justify that the order of the values in our model is realistic. We are mainly interested in the qualitative results such that the exact choice of the values is not so important, as long as we require it to lead to a wildtype yeast cell with 24 division (see also supplementary section S4). We added this to the supplementary section S3.3 and refer to it in the manuscript (l. 206-207).

The higher value of k_2 for cells with a declining repair capacity is surely responsible for lower damage levels, and that is exactly the point we want to make. The higher R the more the effective repair rate will drop during ageing, so these cells only repair faster when they are young. In the case of unlimited repair capacity cells generally have a smaller k_2 but this value remains constant throughout the lifespan. Thus, later on these cells can repair much better and have the advantage of higher effective repair rates. Since we compare cells (that become the founder cells of the populations) that have the same replicative lifespan, we basically investigate when during the life of a cell repair is especially important to influence the health of the lineage. To our knowledge it is not known how much the repair rate is altered during ageing but with our model, and in particular the two biologically distinct parameters k_2 and R, we can still investigate the consequences of it on the population level.

In summary, even though we do not include specific experiments, the model that we use contains biologically interpretable and relevant parameters that are tuned to lead to typical replicative lifespans of individual wildtype yeast cells. We showed that the resulting range of parameters is in accordance with previous computational and experimental work. Furthermore, we can reproduce second-degree rejuvenation on the population level with the model, that was measured experimentally already in the 90s (Kennedy et al., Journal of Cell Biology 1994). We agree that it would be of great value to incorporate further experimental work, especially on the population level, which is however still not completely feasible. But with our model we provide possible measures that could potentially simplify the analysis of cell lineages in the future.

Minor points:

1. There are still some spelling mistakes and misuse of sentence connectors which affect readability. For instance, in the author summary it says: "a model organisms". The subsequent sentence is also strange, particularly the "between how". Could be something like: "...map individual strategies of damage control with properties of the cell population". Also on line 274 from page 9 it says: "populations" rather than "population"

We corrected the spelling mistakes and worked on improving the language in the manuscript.

2. I really don't see the point in introducing the parameter Q in the model. I understand that this parameter was analyzed in previous works by the authors but in the context of this paper it does not seem to serve any purpose and could be incorporated as part of the other terms. If the term is however essential for the authors', it should be justified better why it is included and how it affects the observed results. When it comes to mathematical modelling, simpler models are more powerful and less prone to bias.

We agree that simplifying mathematical models is an important step leading to a more comprehensive model with a reduced number of degrees of freedom. To do so we performed a non-dimensionalisation of the model that resulted in the new parameter Q which cannot be eliminated from the equations. It is an important part of the model that we scale the functional protein content P and the damaged protein content D by their respective threshold values P_c and D_c. Based on that and the model structure we have, it is an inevitable consequence that a new parameter representing the quotient of the two thresholds in one way or another results from the non-dimensionalisation. Consequently, we cannot incorporate Q as part of other parameters and in that way reduce yet another parameter. Borgqvist et al., Scientific reports (2020), presented an extensive analysis of this parameter in a single-cell setting, and estimated a value from experimental data that is used in this work as well. They further showed that given the previously described scaling of P and D there is always Q or $1/Q$ as a new parameter independent of the right hand side of the differential equations. Here, we consider the parameter Q as an essential part of the single-cell model that is in turn the basis of the population model. Since it is however not the focus of this work, we use the estimated parameter from Borgqvist et al., Scientific reports (2020). To emphasise that, we added another paragraph in the supplementary section (end of SI S1) and slightly modified the manuscript (l. 102-107).

3. Bibliography is a little outdated. Specially concerning active quality-control mechanisms in budding yeast. See for instance J. Saarikangas, F. Caudron, R. Prasad, D. F. Moreno, A. Bolognesi, M. Aldea, and Y. Barral, Current Biology 27, 773 (2017), J. Saarikangas and Y. Barral, Elife 4, e06197 (2015). I believe a general review of recent bibliography on the subject together with some minor revisions and some rephrasing (while extending relations to other work) could only improve the paper's impact in the field.

In connection to major points 1 and 2, we included a more detailed and updated description in the introduction of what active and passive quality-control mechanisms can be biologically.

4. Also, I don't see how generating k_1 and k_2 randomly rather than taking them constant impacts positively the results rather than just adding unnecessary noise. Does this change the results qualitatively? If not it could just be put as a comment in the paper with a proper analysis in the appendix. Again, if it is essential it should be justified.

We added the random effects in k1 and k2 to account for a more realistic simulation of cell populations. Many experiments confirm that cells show a high cell-to-cell variability even under the same conditions (Welkenhuysen et al., BMC Systems Biology (2017), Persson et al., Frontiers in Physiology (2020)). However, we agree that it is not particularly necessary to add these effects to draw the same conclusions as we did in this paper. To underline that, we added simulation results without NLME (or equivalently $\sigma = 0$) that show that the results regarding rejuvenation, retention and health do not qualitatively change. Mostly the variability of the single-cell properties is decreased. We describe the new results in the supplementary (SI S7) and further refer to it in the manuscript (l. 232-238), since we think it is a valuable point that shows that our results are valid even under the more realistic assumptions that not all cells have the same parameters. Besides the fact that it is more realistic, our approach also underlines that non-linear mixed effects are a powerful tool to include cell-to-cell variability in models with great potential for future models. We believe that more complicated model structures also show stronger dependency on the heterogeneity in the cell population.

5. There is existing experimental data on the rates of formation of damaged proteins; see for instance J. Saarikangas and Y. Barral, Elife 4, e06197 (2015). This could help reduce the model's degrees of freedom.

We now included experimental justification of the range that k_1 and k_2 can take in the model (SI S3). See also major point 3.

6. Active quality control does not only refer to active transport (page 2). There are other possible mechanisms of damage retention that don't involve active transport of aggregates. See for instance R. Spokoini, O. Moldavski, Y. Nahmias, J. L. England, M. Schuldiner, and D. Kaganovich, Cell reports 2, 738 (2012).

In connection to major point 1 and 2 and minor point 3, we included a more detailed and updated description in the introduction of what active and passive quality-control mechanisms can be biologically.

7. In Fig. 3B. The top two green lines are not clearly explained. What does "intact in mother/daughter mean?"

The intact protein content is the functional protein content P. We changed the label in the figure according to our definition in Box 1 to functional, and added P and D.

8. I am not sure that the assumption of having constant damage retention is very consistent with real-life processes. The choice of this assumption is also not properly justified.

We agree that very likely retention, or more precisely the retention factor re in our model, is not constant throughout a cell's life and it seems reasonable that if the whole functionality declines during ageing, also retention mechanisms would, at least active ones. To our knowledge there is however no experimental data that points towards this assumption. Assuming that the retention factor re is constant is therefore a way to simplify the analysis in the first place. But also since the focus of our work is to see qualitatively what happens if retention mechanisms act in proliferating cells it would not really add to our conclusions. Using an adaptive or declining retention factor would however be a natural continuation of this work to investigate optimal retention strategies.

9. Fig. 3A top seems to reach a plateau, meaning that too much retention does not increase population size.

It is true that it seems like the curve flattens slightly. Under the assumption that the retention factor is constant and a cell needs a minimum amount of functional proteins to live, we cannot increase re even further to confirm that the population size reaches a plateau. In connection to the previous minor point 8 it would be an interesting point to explore adaptive retention to be able to use higher retention factors as well.

10. The term "neglected" in page 2 is a bit strong, perhaps it could be changed to "not considered".

We followed the suggestion and changed the formulation.

Have all data underlying the figures and results presented in the manuscript been provided? Large-scale datasets should be made available via a public repository as described in the PLOS Computational Biology data availability policy, and numerical data that underlies graphs or summary statistics should be provided in spreadsheet form as supporting information.

Reviewer 1: None
Reviewer 2: Yes
Reviewer 3: Yes

PLOS authors have the option to publish the peer review history of their article (what does this mean?). If published, this will include your full peer review and any attached files. If you choose "no", your identity will remain anonymous but your review may still be made public. Do you want your identity to be public for this peer review? For information about this choice, including consent withdrawal, please see our Privacy Policy.

Reviewer 1: No
Reviewer 2: No
Reviewer 3: No
